# mSphere of Influence: Is asymptomatic colonization really silent?

**DOI:** 10.1128/msphere.00250-26

**Published:** 2026-10-07

**Authors:** Brady L. Spencer

**Affiliations:** 1 Department of Microbiology, Immunology, and Cancer Biology, School of Medicine, University of Virginia 174484, Charlottesville, Virginia, USA; Virginia-Maryland College of Veterinary Medicine, Blacksburg, Virginia, USA

**Keywords:** host–pathogen interactions, group B *Streptococcus*, mucosal pathogens, secretion systems, colonization

## Abstract

Brady Spencer is a bacteriologist studying host–pathogen–microbiota interactions. In this mSphere of Influence article, she reflects on how two studies from Scott Hultgren’s laboratory on mucosal imprinting following *Escherichia coli* bladder infection (V. P. O'Brien, T. J. Hannan, L. Yu, J. Livny, et al., Nat Microbiol 2:16196, 2016, http://dx.doi.org/10.1038/nmicrobiol.2016.196; S. K. Russell, J. K. Harrison, B. S. Olson, H. J. Lee, et al., Nat Microbiol 8:875–888, 2023, http://dx.doi.org/10.1038/s41564-023-01346-6) and work by J. C. Whitney, S. B. Peterson, J. Kim, M. Pazos, et al., eLife 6:e26938, 2017, https://doi.org/10.7554/eLife.26938) on T7SSb-mediated interbacterial antagonism shaped her approach to studying group B *Streptococcus* colonization and its potential long-term consequences at mucosal surfaces.

## COMMENTARY

Host–pathogen interactions are often studied in the context of acute infection, but microbes can also exert effects on the host that are subtler and longer-lasting. Hultgren and colleagues demonstrated that *Escherichia coli* infection of the urinary epithelium results in epithelial remodeling (1) and induces epigenetic changes (2) that promote recurrent infection. These studies reframed my thinking of mucosal niches as sites for lifelong host–pathogen–microbiota interactions, the consequences of which may not always be acute or immediately apparent, especially in the context of bacterial colonization.

My laboratory studies the pathobiont group B *Streptococcus* (GBS), a leading cause of neonatal meningitis following vertical transmission during pregnancy. As GBS colonization of the female genital tract is largely considered asymptomatic (3), the justification for its study has largely been that it is a prerequisite for neonatal invasive disease and that it contributes to some adverse pregnancy outcomes. However, framing asymptomatic colonization as “silent” or lacking impact may not be accurate. Longitudinal studies have shown that GBS can be detected throughout pregnancy (4), and those colonized with GBS in their first pregnancy are more likely to be colonized in subsequent pregnancies (5). Because GBS is a persistent colonizer, it is possible that its chronic presence may impact the mucosa, even if that impact is not acute or pathological.

Beyond the vaginal tract, vertical transmission also results in GBS colonization of the neonatal gastrointestinal tract (6–9). Microbial exposure in early life, during a critical period known as the “window of opportunity,” is known to have long-term consequences on immunity and development (10, 11). For example, asymptomatic *Clostridioides difficile* colonization of the infant GI tract was recently shown to impact the development of the intestinal epithelium and mucosal innate immunity, with these alterations persisting later in life (12). Despite the growing body of work supporting the importance of host–pathogen interactions in early life, the impact of GBS colonization on the neonatal GI tract and long-term outcomes remains virtually unknown.

One mechanism by which bacteria interact with the host and neighboring bacteria is through expression of secretion systems that export effector proteins. The type VII secretion system (T7SS) is a specialized protein secretion system first characterized in mycobacteria and since identified in Bacillota (13, 14). In gram-positive bacteria, T7SSb exports effector proteins and toxins that mediate interbacterial competition and interact with host cells. When I began investigating this system in 2019 as a postdoc, the field of T7SSb in gram-positive organisms was still relatively new. Work in *S. aureus* was pioneered by Dominique Missiakas (15–17) and Tracy Palmer (18–21), and John Whitney, Joseph Mougous, and colleagues had recently described a broadly distributed family of polymorphic toxins exported by T7SSb that mediate contact-dependent antagonism between gram-positive bacteria, including in *Streptococcus intermedius* (22). This work suggested that T7SSb-exported effectors could shape interbacterial interactions, which may have broader implications for the microbial community. This led me to wonder how the T7SSb might influence GBS interactions within complex microbial communities. Finding that essentially all whole-genome sequences of human GBS isolates available on GenBank (circa 2020) encoded a version of this system led me to pursue the GBS T7SSb and its impact on colonization and disease for the remainder of my postdoctoral work and as the basis of my independent research program (23–26). A major focus of our current work centers on how GBS T7SSb effectors impact the microbiota, immune response, and/or epithelial barriers during colonization of the vaginal tract and neonatal GI tract, as well as the potential long-term consequences of these interactions.

Individual events with lasting impact have also shaped the motivation for my career and what I hope my lab achieves over time. My childhood best friend passed away during my PhD from an infectious disease not unlike that which I was studying in my dissertation research. During my postdoc studying group B *Streptococcus* pathogenesis, two friends of friends lost their babies due to adverse pregnancy outcomes of bacterial etiology. In the past year alone, I sat next to two women on airplanes who, after learning what my lab studies, shared with me that they too had lost babies to bacterial infections during pregnancy. These encounters remind me that there are real people behind our research questions. Like most scientists, I love the challenge of basic research, making discoveries, and advancing fundamental knowledge, but these experiences have shaped how I think about our work and serve as my motivation for the days when the work is difficult. Even if most of our research does not have direct clinical translation, my hope is that our small scientific contributions over time will have long-term impact.
